# A Rare Case of Immune‐related Adverse Events Localized to the Small Intestine

**DOI:** 10.1002/deo2.70187

**Published:** 2025-09-07

**Authors:** Ryo Morikawa, Ryosuke Kanaya, Hiromichi Shimizu, Masayoshi Fukuda, Yasuhiro Nemoto, Toshimitsu Fujii, Towako Taguchi, Kurara Yamamoto, Kazuo Ohtsuka, Ryuichi Okamoto

**Affiliations:** ^1^ Department of Gastroenterology and Hepatology Institute of Science Tokyo Japan; ^2^ Division of Comprehensive Pathology, Institute of Science Tokyo Japan; ^3^ Division of Human Pathology, Institute of Science Tokyo Japan

**Keywords:** endoscopy, immune checkpoint inhibitor, immune‐related adverse events, prednisolone, small intestine

## Abstract

Enterocolitis is a common gastrointestinal manifestation of immune‐related adverse events (irAEs); however, only a few studies have reported on irAE enteritis with localized active inflammation in the small intestine. Here, we report the case of a 74‐year‐old man who developed diarrhea, abdominal pain, and oral intake difficulty and was subsequently hospitalized after receiving atezolizumab for pulmonary adenocarcinoma. Computed tomography and enterocolonoscopy revealed active inflammation in the small intestine but not in the colon, leading to the final diagnosis of irAE enteritis. After initiating prednisolone at a dose of 60 mg/day, his symptoms improved rapidly, and a follow‐up enterocolonoscopy revealed a marked reduction in inflammation. Being a relatively rare gastrointestinal toxicity, irAE enteritis often goes unrecognized due to diagnostic challenges, but can lead to serious AEs such as perforation. Therefore, even if colonoscopy findings are normal, a thorough examination of the small intestine is essential for patients who develop gastrointestinal symptoms while undergoing immune checkpoint inhibitor therapy. We herein report a rare case of irAE enteritis confirmed through endoscopic and pathological examination, which has not been previously reported.

## 1 Introduction

In recent years, immune checkpoint inhibitors (ICIs) have been used to treat various cancers [1], demonstrating high efficacy and improved prognosis. However, immune‐related adverse events (irAEs), resulting from the breakdown of immune tolerance, pose an obstacle to continuing cancer treatment. One of the most commonly associated AEs of ICI treatment is gastrointestinal toxicity [2]. Although enterocolitis is a common manifestation of irAE‐related gastrointestinal toxicity, to the best of our knowledge, only a few studies have reported on irAE enteritis with localized active inflammation in the small intestine, and no studies have reported on endoscopic and pathological examination findings. Although irAE enteritis is a less frequent gastrointestinal toxicity that often goes unrecognized due to diagnostic difficulties, it may cause serious AEs such as perforation. Here, we present a case of irAE enteritis, along with a review of the relevant literature.

## 2 Case Report

A 74‐year‐old man with a history of ileocecal resection (for unknown reasons) began treatment with atezolizumab at a dose of 1200 mg/body every 3 weeks for pulmonary adenocarcinoma at the Department of Respiratory Medicine in our hospital. After 7 months, due to progression of the primary disease, atezolizumab was discontinued after the 10th course, and treatment was switched to TS‐1. However, TS‐1 was discontinued 1 month later as the patient experienced decreased appetite and diarrhea (once daily) that worsened to five times daily, accompanied by abdominal pain and impaired oral intake. He had not been taking non‐steroidal anti‐inflammatory drugs. On presentation to the hospital, the patient had a temperature of 37.6°C and exhibited tenderness in the lower abdomen, while rebound tenderness was absent. Laboratory examination revealed anemia (hemoglobin 10.9 g/dL), elevated C‐reactive protein (1.54 mg/dL), hypoalbuminemia (albumin 2.8 g/dL), and impaired renal function (creatinine 3.94 mg/dL), whereas thyroid function test results showed no abnormalities.

**FIGURE 1:**
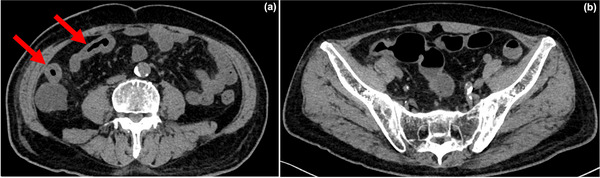
(a) and (b) Images of abdominal computed tomography prior to prednisolone treatment. Computed tomography revealed wall thickening in the small intestine but no significant abnormalities in the colon.

**FIGURE 2:**
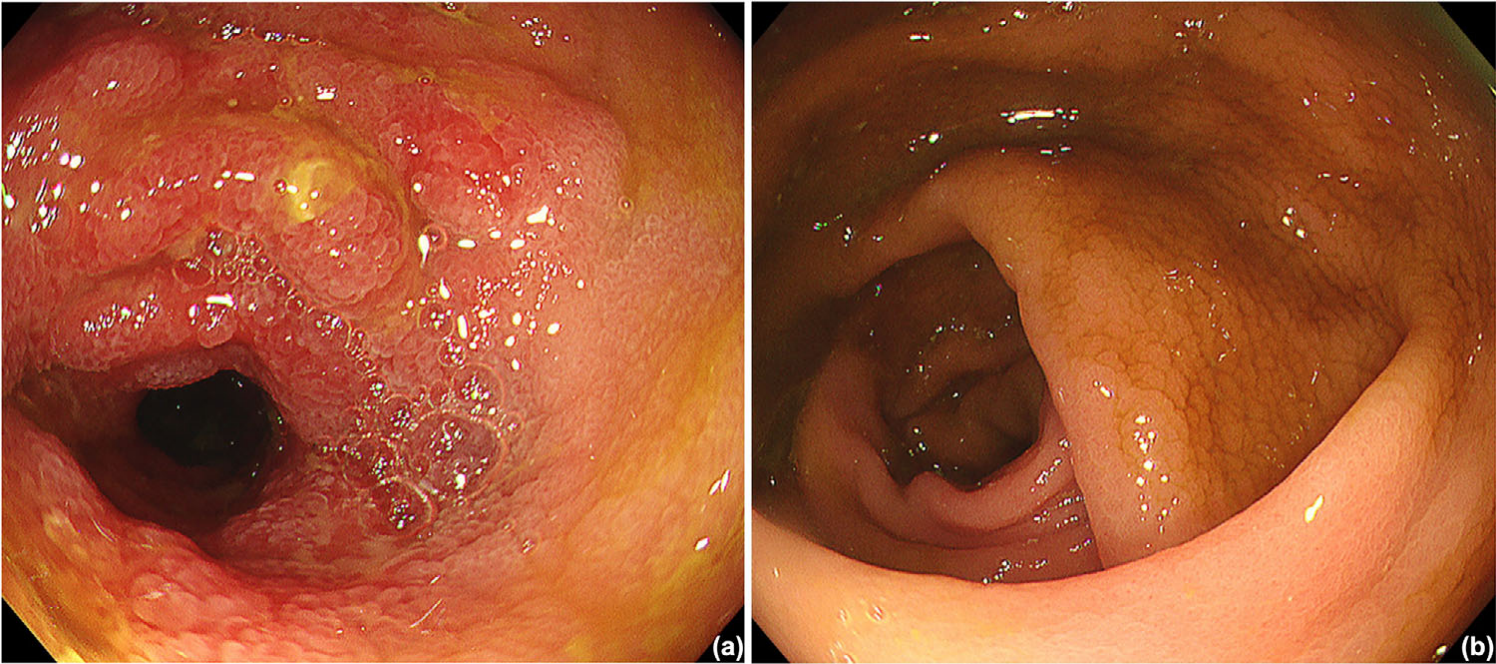
Endoscopic images of (a) small intestine and (b) colon prior to prednisolone treatment. (a) Endoscopy showed mucosal edema, erythema, and scattered erosions, and atrophied villi in the small intestine (b) and mucosal edema without any erosions or ulcers in the colon.

**FIGURE 3:**
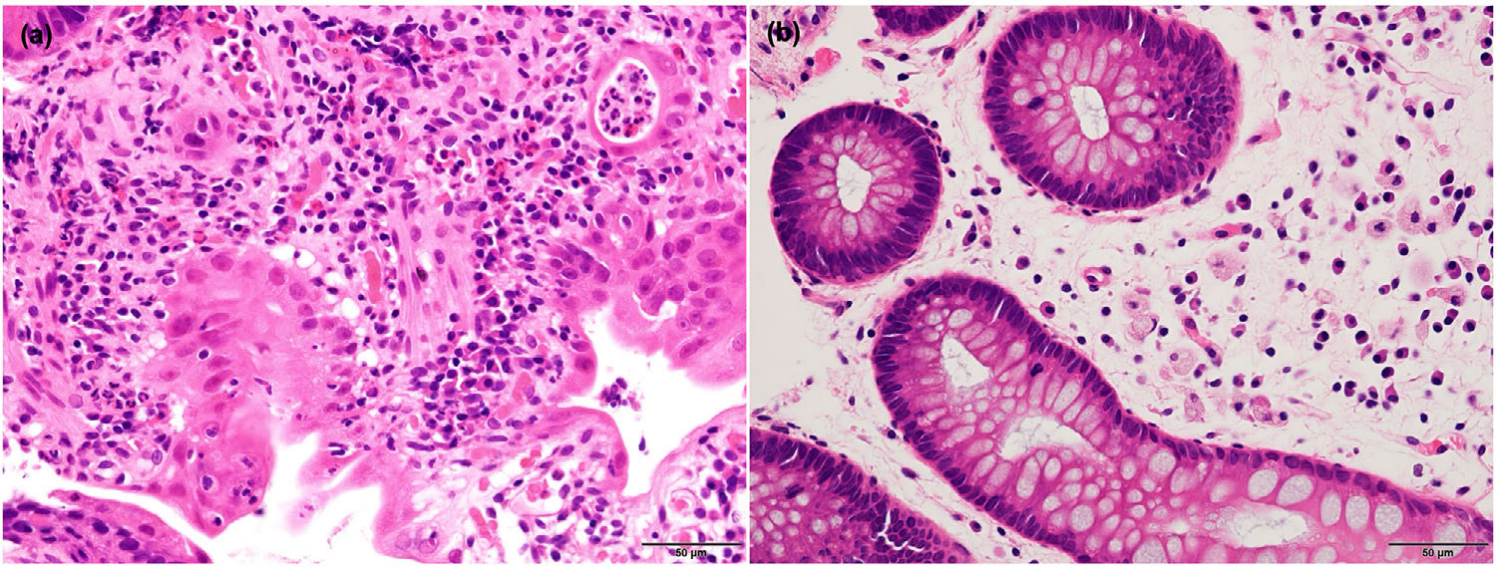
Histopathological findings of hematoxylin and eosin staining prior to prednisolone treatment for (a) small intestine and (b) colon. The small intestine showed inflammatory cell infiltration in the lamina propria and crypt abscesses, with no apoptotic features. In contrast, the colonic mucosa showed no active inflammatory changes.

The patient was admitted emergently after being diagnosed with diarrhea, enteritis, and acute kidney injury of unknown etiology. Although the patient was placed on a fast, received intravenous fluid therapy, and was not administered TS‐1, his symptoms did not improve. Computed tomography (CT) revealed wall thickening in the small intestine, suggesting enteritis, while showing no significant abnormalities in the colon (Figure 1). Endoscopy revealed edema, erythema, scattered erosions, atrophied villi, and ulcers in the ileal mucosa (Figure 2) as well as edema with no erosions or ulcers in the colonic mucosa. Histopathological examination of the small intestine showed villous shortening, crypt abscesses, and infiltration of neutrophils and mononuclear cells into the lamina propria, without prominent apoptosis (Figure 3), whereas that of colonic mucosa showed no active inflammatory changes. Stool cultures, *Clostridium difficile* toxin and antigen tests, and cytomegalovirus nucleic acid testing yielded negative results.

**FIGURE 4:**
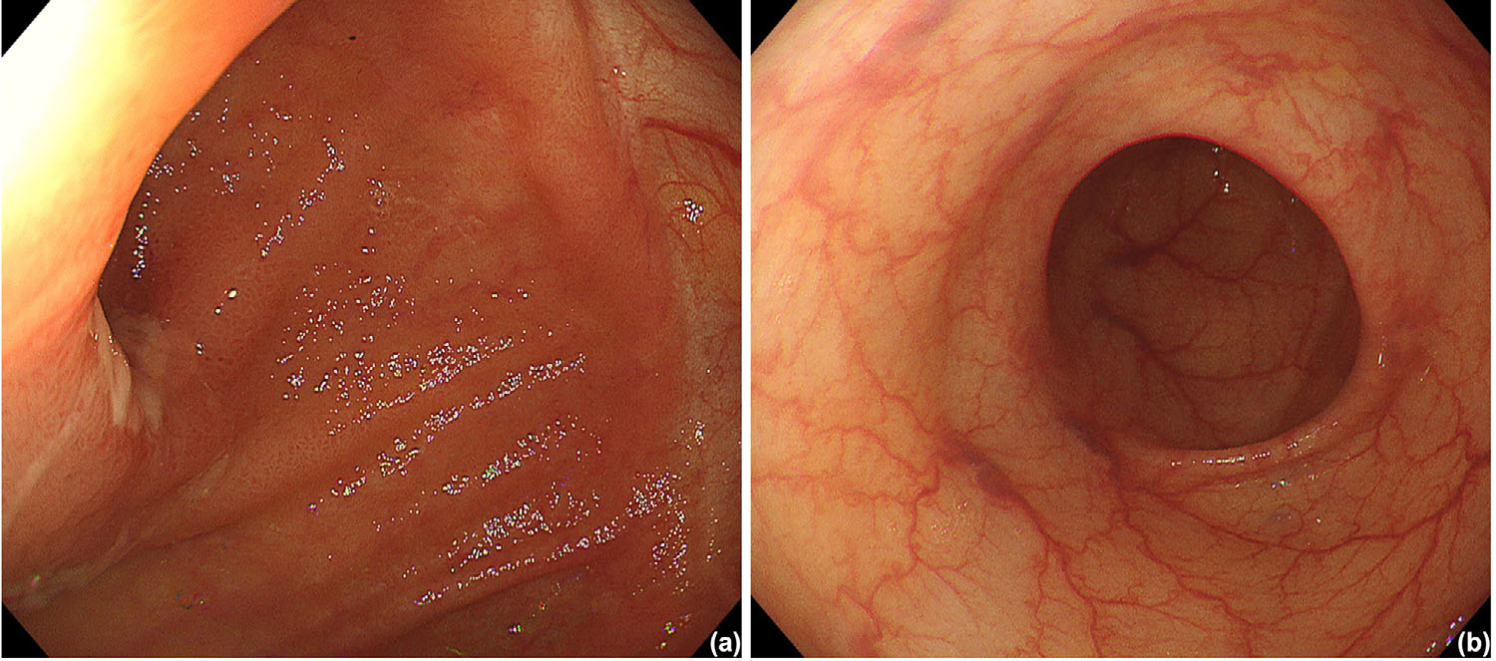
Endoscopic images of (a) small intestine and (b) colon after prednisolone treatment. Endoscopy showed improvement in the inflammatory findings in the small intestine and no edema in the colon.

Based on the clinical, radiological, endoscopic, and histopathological findings, he was diagnosed with Common Terminology Criteria for Adverse Events grade 2 irAE enteritis and was administered prednisolone at a dose of 60 mg/day. Clinical symptoms such as diarrhea and abdominal pain were mitigated, and the blood test results also improved. Prednisolone was tapered every 2 weeks and discontinued over a total period of 14 weeks. During this time, no symptom relapse was observed, and the C‐reactive protein level decreased to 0.72 mg/dL. A follow‐up colonoscopy performed 3 months after the treatment initiation showed improvement in the inflammatory findings (Figure 4 ). While initiating immunosuppressive therapy was considered as a treatment option to achieve mucosal healing, considering the improvement in his abdominal symptoms, the decline in his performance status, and the absence of plans to resume treatment with ICIs, additional therapy was not initiated.　The irAE enteritis did not relapse during the follow‐up period of >6 months.

## 3 Discussion

Although ICIs have shown remarkable efficacy and are expected to continue playing a significant role in cancer treatment, the rate of treatment discontinuation due to irAEs has been reported to be approximately 3%–7.1% in clinical trials [3]. Gastrointestinal toxicity is considered one of the most common irAEs. According to a previous report, 19.2% of patients treated with programmed cell death protein 1 (PD‐1) or programmed death‐ligand 1 (PD‐L1) inhibitors developed diarrhea, and 1.3% developed colitis [2]. In another report, treatment discontinuation due to gastrointestinal toxicity occurred in 6.1% and 5.4% of patients receiving PD‐1 and PD‐L1 inhibitors, respectively [4]. Most cases are enterocolitis, but irAE enteritis, as observed in the present case, is a rare AE. A previous retrospective cohort study reported that among the 137 patients with lung cancer who had undergone CT imaging before and after ICI treatment, 16 had colitis and/or enteritis, whereas only one had enteritis without colitis [5]. In another study, no cases of isolated enteritis were identified among the 53 patients with lung cancer (colitis: *n* = 7; enterocolitis: *n* = 2) who received ICI treatment and had baseline CT imaging available [6]. A PubMed search using the terms “irAE,” “enteritis,” “without,” and “colitis” yielded two cases (Table 1) [7, 8], which had CT findings indicating inflammation in the small intestine, with no active inflammatory findings in the colon. However, neither case was evaluated endoscopically or pathologically to confirm the absence of colonic inflammation. Both these types of evaluations are necessary to confirm microscopic‐type colitis, which is identified in some cases of ICI‐related colitis. In this case, we confirmed the diagnosis of enteritis without active colitis both endoscopically and histologically (Figure 2, 3).

**TABLE 1:** Previously reported cases of immune‐related adverse event (irAE) localized to the small intestine.

Year	Author	Age/sex	ICIs	Time of onset	Prognosis
2018	Abdulnabi	52/F	Ipilimumab + nivolumab	N/A	Surgery
2019	Beck	62/F	Pembrolizumab	8 weeks	Surgery

Regarding the distribution of irAE enteritis with localized active inflammation in the small intestine, one of the two cited case reports [7, 8] involved the ileum, while the other involved both the jejunum and ileum. In our case, CT imaging showed the most prominent inflammation in the ileum.

Endoscopic findings of irAE‐related enteritis have been reported to include atrophied villi, friable mucosa, mucosal shedding, erythema, erosion, and ulcers^S1^. In the present case, atrophied villi, erosions, and ulcers were also observed.

IrAE enteritis has been reported to result in severe complications such as perforation. A PubMed search using the terms “irAE,” “enteritis,” and “perforation” yielded three cases: Two cases showed CT findings of small intestinal inflammation with signs of peritoneal irritation [7, 8], and one case showed CT findings of edematous jejunum and colon, but colonoscopy showed no erosions or ulcers. The patient subsequently developed perforation before initiating treatment [9]. In the present case, CT imaging revealed thickening of the ileal wall, strongly suggesting small intestinal inflammation. Although endoscopy revealed no significant inflammation in the colon, further evaluation extending into the small intestine confirmed the diagnosis of enteritis. In this case, we believe that a quick diagnosis and prompt initiation of prednisolone therapy prevented severe potential complications.

Systemic corticosteroids are the first‐line treatment for Common Terminology Criteria for Adverse Events grade ≥2 irAE enterocolitis [10]. If there is no response within 72 h or if symptoms relapse during corticosteroid tapering, a transition to second‐line immunosuppressive therapy should be considered. In addition, if endoscopic evaluation for treatment response shows incomplete mucosal healing, continued immunosuppressive therapy is recommended. In our case, symptoms improved within a few days of initiating corticosteroid treatment, and no recurrence was observed after tapering the corticosteroid. Although the follow‐up colonoscopy showed incomplete mucosal healing, additional immunosuppressive therapy was not considered due to the patient's improved abdominal symptoms, decreased performance status, and the absence of plans to resume ICIs.

We herein present a rare case of irAE enteritis. In patients who develop persistent gastrointestinal symptoms during ICI therapy, even when colonoscopy reveals no abnormalities, it is important to evaluate the entire gastrointestinal tract using cross‐sectional imaging and to consider the indication for small bowel endoscopy.

We believe this report provides valuable insights into the diagnostic approach for irAE‐related gastrointestinal toxicities.

## Conflicts of Interest

The authors declare no conflicts of interest.

## Supporting information



Supporting File: deo270187‐sup‐0001‐SuppMat.docx

## Data Availability

Information about this report is available from the corresponding author upon request.
