# Supplementary material for: A Rare Case of Immune‐related Adverse Events Localized to the Small Intestine
Source: DEN Open. 2025 Sep 7;6(1):e70187. doi: 10.1002/deo2.70187 (PMC12415260; doi:10.1002/deo2.70187)
Supplement: Supplementary file 1 — Supporting File: deo270187‐sup‐0001‐SuppMat.docx [file DEO2-6-e70187-s001.docx]

Supplementary Material

References for Case Reports

a.Hayashi Y, Hosoe N, Takabayashi K, et al. Clinical, Endoscopic, and Pathological Characteristics of Immune Checkpoint Inhibitor-Induced Gastroenterocolitis. Dig Dis Sci 2021; 66: 2129–34.

b.Gonzalez RS, Salaria SN, Bohannon CD, et al. PD‐1 inhibitor gastroenterocolitis: case series and appraisal of ‘immunomodulatory gastroenterocolitis.’ Histopathology 2017; 70: 558–67.
